# Integrating an AI platform into clinical IT: BPMN processes for clinical AI model development

**DOI:** 10.1186/s12911-025-03087-4

**Published:** 2025-07-02

**Authors:** Kfeel Arshad, Saman Ardalan, Björn Schreiweis, Björn Bergh

**Affiliations:** 1 Institute for Medical Informatics and Statistics, Kiel University and University Hospital Schleswig-Holstein, Kiel, Germany; 2 Medical Data Integration Center, University Hospital Schleswig-Holstein, Kiel, Germany

**Keywords:** Artificial intelligence, Machine learning, AI platform, Healthcare, BPMN, Process diagrams, Clinical IT, Medical data integration center

## Abstract

**Background:**

There has been a resurgence of Artificial Intelligence (AI) on a global scale in recent times, resulting in the development of cutting-edge AI solutions within hospitals. However, this has also led to the creation of isolated AI solutions that are not integrated into clinical IT. To tackle this issue, a clinical Artificial Intelligence (AI) platform that handles the entire development cycle of clinical AI models and is integrated into clinical IT is required. This research investigates the integration of a clinical AI platform into the clinical IT infrastructure. This is demonstrated by outlining the stages of the AI model development cycle within the clinical IT infrastructure, illustrating the interaction between different IT system landscapes within the hospital with BPMN diagrams.

**Methods:**

Initially, a thorough analysis of the requirements is conducted to refine the necessary aspects of the clinical AI platform with consideration of the individual aspects of clinical IT. Subsequently, processes representing the entire development cycle of an AI model are identified. To facilitate the architecture of the AI platform, BPMN diagrams of all the identified processes are created. Clinical use cases are used to evaluate the processes using the FEDS framework.

**Results:**

Our BPMN process diagrams cover the entire development cycle of a clinical AI model within the clinical IT. The processes involved are Data Selection, Data Annotation, On-site Training and Testing, and Inference, with distinctions between (Semi-Automated) Batch Inference and Real-Time Inference. Three clinical use cases were assessed to evaluate the processes and demonstrate that this approach covers a wide range of clinical AI use cases.

**Conclusions:**

The evaluations were executed successfully, which indicate the comprehensive nature of our approach. The results have shown that different clinical AI use cases are covered by the BPMN diagrams. Our clinical AI platform is ideally suited for the local development of AI models within clinical IT. This approach provides a basis for further developments, e.g., enabling the training and deployment of an AI model across multiple sites or the integration of security- and privacy-related aspects.

**Supplementary Information:**

The online version contains supplementary material available at 10.1186/s12911-025-03087-4.

## Background

In the past several years, we saw a reemergence of Artificial Intelligence (AI) around the world [1, 2]. With the rise of AI, AI algorithms are used more frequently in many domains [3, 4]. There is also a considerable development of AI-based systems in healthcare settings [5, 6]. In several medical domains, such as radiology [7], cardiology [8], dermatology [9] or pathology [10], we already see progress in clinical decision support using AI systems in recent years. Despite extensive progress in the development of AI systems in many medical domains [11], translation into clinical routine is still in its early stages and holds many challenges [12]. One major challenge is that in many clinical settings there are only isolated AI solutions that are not fully integrated into the existing clinical IT [13] or data need to be distributed across multiple IT systems to enable AI support in several domains. Initial approaches for integrating a clinical AI platform into the clinical IT were already highlighted, although they are intended for specific medical use cases or individual data types such as alphanumeric data [14] or imaging data [15, 16].

A more comprehensive clinical AI platform solution that covers the entire spectrum of clinical IT landscapes is currently not available. One factor hampering this is the clinical source systems’ focus on documentation, billing and archiving clinical data [17]. Overcoming this important barrier is the aim of approaches like medical data warehousing, clinical data repositories and knowledge management platforms. As part of the German Medical Informatics Initiative^1^, all German University Hospitals are currently rolling out so called (medical) data integration centers (MeDICs/DIZ) [18, 19]. The main idea of these MeDICs is to integrate clinical routine data from the respective University Hospital’s electronic medical record (EMR) system, cleanse, harmonize and make them discoverable and usable for research purposes in particular, for secondary use in general. MeDICs are not only setting up IT infrastructures but also an organizational structure including processes on how to request, access and use these data [18, 19].

Due to the MeDICs, real-world data are readily available and the secondary use of data is possible. Requested data from a MeDIC cannot only be used for retrospective biomedical research but also for other purposes like training AI models and subsequently using them for inference. To enable broad clinical application of AI, it is necessary that a clinical AI platform is also integrated into the existing clinical IT landscapes for clinical decision support.

## Objectives

To ensure the successful integration of a clinical AI platform, it is necessary to map out the processes involved in the development and application of clinical AI models. Therefore, it is important to define target processes that are relevant from the perspective of stakeholders in the clinical domain. The objective of this study is to describe these processes using Business Process Model and Notation (BPMN) diagrams, thus laying the foundation for developing the architecture of a clinical AI platform.

## Methods

In the following, we describe our setting, the requirements analysis methods, the FEDS framework and the use cases for evaluation.

### Setting

We performed our project at the University Hospital Schleswig-Holstein (UKSH), which is the second-largest hospital in Germany, the only university hospital and maximum care provider in the state of Schleswig-Holstein (northern Germany). Two IT system landscapes can be distinguished within UKSH. First, there are the primary systems, which are used to store all patient treatment-related data, for example, the electronic medical record (EMR) system and the picture archiving and communication system (PACS). The EMR system contains all the medical documentation about patients treated within a healthcare organization [20]. Hospitals, general practitioners, doctors’ offices, rehabilitation organizations or care organizations are considered healthcare organizations. A PACS system is used to acquire, transmit, display and archive medical imaging data [21]. The second IT system landscape consists of the research IT systems. They include all kinds of research databases and registries, e.g., Excel sheets and Access databases. Furthermore, electronic data capture (EDC) systems for structured research documentation are part of the research IT landscape. Additionally, biomaterial information systems to manage biomaterials and their information exist.

The UKSH MeDIC integrates the IT of patient treatment and research. Data from the EMR and subsystems like PACS, laboratory information system, but also research databases, study documentation and biomaterial information are integrated, harmonized and annotated within the MeDIC platform. Additional systems include a Master Patient Index to manage demographic data and a consent management system to process and enforce patient consents [18, 19].

The KI-SIGS project aims at building an AI space for intelligent health systems, which consists of three components: (1) development of four platform projects (a-d) dealing with (a) collaboration, (b) technical aspects, (c) regulatory aspects and (d) responsible innovation, (2) development of nine clinical use cases and (3) improving the healthcare ecosystem in northern Germany [22]. This project is a collaboration of universities, university hospitals and companies in northern Germany. One of the four platform projects in KI-SIGS is the “Technical AI Platform” (b), which designs a clinical AI platform that can obtain and process data to train and subsequently deploy AI models. Additionally, the Clinical AI platform integrates into the existing IT system landscapes of UKSH. Furthermore, there are nine clinical use cases which are referred to as application and innovation (A&I) projects. Each of the A&I projects is working on applying state-of-the-art machine learning (ML) methods on a specific clinical use case. These use cases employ a broad variety of areas where ML can be utilized, from analysis of patients’ vital data produced by medical devices at the ICU [23] to medical image analysis [24]. The output of the AI platform project includes the requirements analysis for identifying and documenting the needs as well as objectives for developing a clinical AI platform. The requirements analysis serves as the foundation for the subsequent steps of development. Subsequently, a system architecture of the AI platform is to be designed that maps the development cycle of an AI model derived from the BPMN diagrams. Conclusively, a proof of concept will be developed based on the system architecture of the clinical AI platform, which specifies the main functionalities of the AI platform. This involves creating a functional prototype to validate its feasibility and demonstrate its core functionalities. The proof-of-concept provides valuable insights for further refinement and development of a productive clinical AI platform.

### Requirements analysis

Prior to the creation of the BPMN diagrams, we conducted a requirements analysis, which forms the basis of the BPMN processes.

For our proposed AI platform, we extracted functional and non-functional requirements from different stakeholders in an iterative manner to ensure their consistency, completeness, and correctness. First, we conducted semi-structured interviews [25] by asking all stakeholders about functional and non-functional requirements. Accordingly, the focus was on the discovery of requirements relevant to the AI platform. Two stakeholder groups were identified that cover the entirety of the AI platform requirements. The first stakeholder group consists of computer scientists. In this case, both the computer scientists working in the AI platform project and the computer scientists working on the application (A&I) projects in KI-SIGS were interviewed. There were 7 computer scientists working on the AI platform and 28 computer scientists working on the application projects. Thus, a total of 35 stakeholders working in clinical computer science were interviewed. The second stakeholder group consists of clinical physicians. Within the AI platform, one clinical physician was interviewed. Within the A&I projects, a total of 7 clinical physicians were interviewed. Accordingly, a total of 8 clinical physicians were interviewed. Starting with the semi-structured interview, we performed the conventional steps for a requirements analysis [26]. First, the requirements were identified and gathered. We consequently extracted the main demands for an AI platform and current limitations. The requirements were initially gathered in a mind map for the collection process. Then the requirements were reviewed and categorized by the stakeholders, who are working on the AI platform. The review of the requirements mainly included targeting and plausibility checks. Subsequently, the requirements were further categorized as functional and non-functional requirements. The entire outlined process was performed in three iterations with all stakeholders involved in the development of the AI platform. Then the requirements were documented and are now managed in a cloud to which all project participants have access.

The development and deployment of clinical AI models can be represented by consecutive processes [27, 28]. Based on the processes outlined by Lu et al. [28], we have identified four overarching use-case-agnostic processes that cover the complete workflow for the development and deployment of clinical AI models. For this purpose, we selected the processes outlined by Lu et al. [28] after the positive decision of clinical usefulness and feasibility and integrated clinical validation and model testing into the training process, as these are strongly interrelated according to Lu et al. [28].

The main functional requirements that represent the necessary boundary conditions for our process flows are outlined in Table 1. These requirements can be derived from the functional requirements for medical data integration into knowledge management environments [29]:

**Table 1:** Main functional requirements for development of the BPMN diagrams

1. Infrastructural conditions are provided such that the AI platform can run on-premises and thus no data leave the site.
2. The AI models should be stored together with associated descriptive information in the AI platform or in the knowledge management platform.
3. The AI platform should be able to store the data temporarily.
4. The AI platform should be linked to the medical data integration center and hospital information system.
5. The AI platform serves as a data mart and should process a subset of the data from the medical data integration center.
6. The AI platform should support alphanumeric data, including structured, semi-structured and unstructured data and multimedia data.
7. The AI platform should be able to process anonymized, pseudonymized and non-pseudonymized data.
8. The AI platform should be designed to be able to be connected to other data platforms.
9. The AI platform should support all identified use-case-agnostic processes.

In order to validate the requirements outlined in Table 1, which serve as the boundary conditions for our BPMN diagrams, we applied the validation method proposed by Odeh [30]. This involved an inspection of the requirements by the stakeholders, with particular attention paid to the 4Cs (correctness, completeness, consistency and clarity). Additionally, we ensured that the requirements were necessary, testable and designable.

### FEDS framework

We evaluated the BPMN diagrams using the Framework for Evaluation in Design Research (FEDS) framework [31]. The FEDS framework was developed to provide a strategy for the evaluation of Design Research Science (DSR) projects. DSR can be defined as: “a problem-solving paradigm that seeks to enhance human knowledge via the creation of innovative artifacts“ [32]. FEDS contains a two-dimensional characterization for the evaluation of DSR projects. One dimension includes the functional purpose of the evaluation, whether it is formative or summative. The second dimension identifies the paradigm of the evaluation, whether it is artificial or naturalistic. The FEDS evaluation consists of four steps: stating the objectives of the evaluation, selecting the evaluation strategy, identifying the attributes to be evaluated and designing the evaluation episodes.

First, the objective of the evaluation is determined. The goal for our evaluation is to minimize the technical uncertainties and risks in the future integration and development of the clinical AI platform. We then select the strategy for the evaluation based on the goal. Since we want to determine whether the technical aspects work as intended in our BPMN diagrams and it is a highly technically oriented design, we apply the Technical Risk & Efficacy strategy. This strategy is designed to mitigate technical risk by evaluations that are done in a controlled and simulated environment to identify potential issues at an early stage, prior to implementation. This is a reasonable approach when the potential for errors during implementation could result in significant costs. We then check which attributes should be evaluated. Here, we choose the accuracy of the process representation as the main attribute. Accordingly, the evaluation should show the completeness of the development process of an AI model in our BPMN diagrams. Subsequently, we define three evaluation episodes to demonstrate the completeness of the BPMN diagrams. In the following, we introduce these evaluation episodes in the form of three use cases that represent a broad range of AI applications in the clinical domain.

### Use cases

We selected three clinical use cases for the evaluation of the BPMN diagrams. The first use case for our evaluation is from the KI-SIGS project and is referred to as “digital x-ray assistant”. This use case is about assessing the quality of upper ankle radiographs. In this use case radiographs of the upper ankle joint are rated from 1 to 3 by radiologists. 1 stands for excellent image quality, 2 stands for acceptable image quality, while 3 stands for non-acceptable image quality. The aim of this use case is to train an AI model that can perform this assessment. In this case, it is the training of a Convolutional Neural Network (CNN) [33, 34].

The second use case for evaluation is the distinction between benign and malignant tumors in breast carcinoma. The Wisconsin Breast Cancer data set [35] can be used for this purpose. The data set consists of structured pathology reports (alphanumeric data) describing the cell nucleus from digitized images of a breast cancer. For example, the mean value of the distances from the center to the points on the perimeter is calculated and recorded as a numerical value. There are 10 features in total. In addition, for each data instance, it is indicated whether the breast cancer is benign or malignant. Accordingly, the data are already annotated and do not require further annotation. The goal is to train an AI model that can predict whether it is a benign or malignant breast cancer using new input data. In this case, the AI algorithm is a decision tree model with non-correlated features as input parameters [36], which should distinguish whether the input data are indicative of a malignant or benign tumor. Therefore, the output can either represent benign or malign.

The third use case for evaluation is also from the KI-SIGS project and is referred to as “Risk indicators for cardiopulmonary decompensation in intensive care units by monitoring vital signs” [23]. The risk assessment is achieved through a scoring system designed to rank and predict pulmonary and hemodynamic decompensation based on vital parameters. A Gated Recurrent Unit (GRU) network is used for the training. The vital parameters, such as heart rate or oxygen saturation, are used as input parameters. A complete list of the input parameters can be found in [23].

## Results

The BPMN diagrams include the processes of Data Selection, Data Annotation, On-Site Training and Testing and Inference, whereas for Inference a case distinction was made between (Semi-Automated) Batch Inference and Real-Time Inference.

In the context of BPMN, we define three sub-pools within UKSH: EMR, MeDIC and the Clinical AI Platform. The MeDIC includes two lanes: *Repository*, which holds the entire medical data within MeDIC and *Mart*. A mart is a specific cohort data set. The Clinical AI Platform includes two lanes: *Front-End* and *Application*. The *Application* lane includes technical subcomponents such as a *Data Broker*, which acts as an API between the Clinical AI Platform and the other IT system landscapes; an *AI Processing Unit*, which is responsible for compiling the data cohort, performing data annotation and training, and orchestrating the associated processes; a *Model Repository*, which contains the trained models; and a *Repository for Temporary Data*, which serves as temporary data persistence for the cohort data.

### Data Selection

**Fig. 1:**
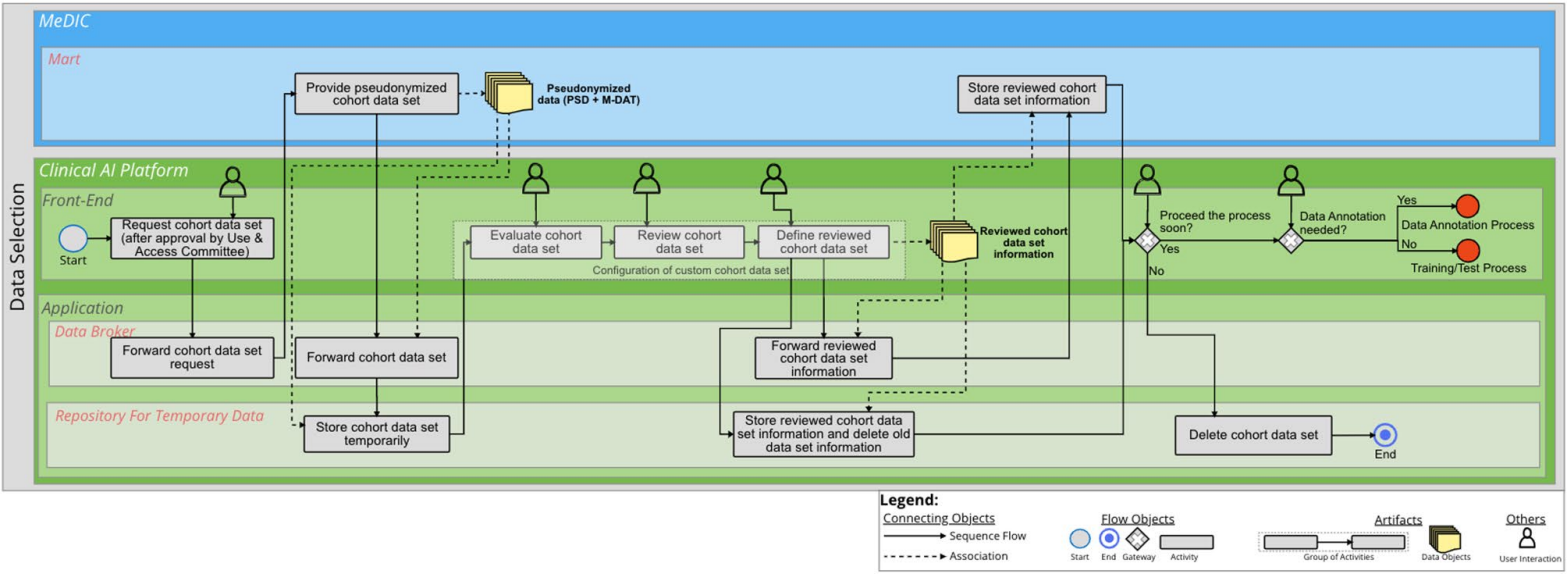
BPMN diagram for Data Selection. Two IT system landscapes are illustrated here as sub-pools: the MeDIC and the Clinical AI Platform. For the MeDIC, the lane for the *Mart* and the lanes for the *Front-End* and *Application* of the Clinical AI Platform are shown here. The technical sub-components of *Data Broker* and the *Repository for Temporary Data* are relevant in the *Application* lane for this process

The first process is Data Selection. This process focuses on selecting a suitable cohort and its corresponding data set for a specific AI use case, e.g., laboratory data or imaging data from radiology. In this case, it is important to note that we assume that the data cohort is already approved for use by the Use & Access Committee and then prepared by the MeDIC’s data transfer unit. The user can subsequently select an already pseudonymized cohort data set in the *Front-End*, which is made available as a mart in the MeDIC. The request is forwarded to the MeDIC via the *Data Broker*. During this process, both alphanumeric and multimedia data can be made available from the MeDIC *Repository* in the MeDIC *Mart*. Then, the data are stored in the *Repository for Temporary Data* via the *Data Broker*. The user evaluates and verifies the pseudonymized data. Afterwards, the cohort data set is defined by selecting adequate data from the provided mart that meets the requirements of the specific AI use case. In the next step, the information about the defined cohort data set is forwarded by the *Data Broker* to the MeDIC *Mart* and the *Repository for Temporary Data*, while the initial cohort data set is deleted. In the *Front-End*, the user is shown that the storage of the defined cohort data set was successful and in addition, the user is asked whether the process should be continued. In the case of continuation, the user can either continue with the Data Annotation process or, if no annotation is required, continue with the On-site Training and Test process. Otherwise, the data are deleted from the Repository for Temporary Data. The BPMN diagram for Data Selection is illustrated in Fig. 1. An additional file shows the diagram in a higher resolution [see Additional file 1]’.

### Data Annotation

**Fig. 2:**
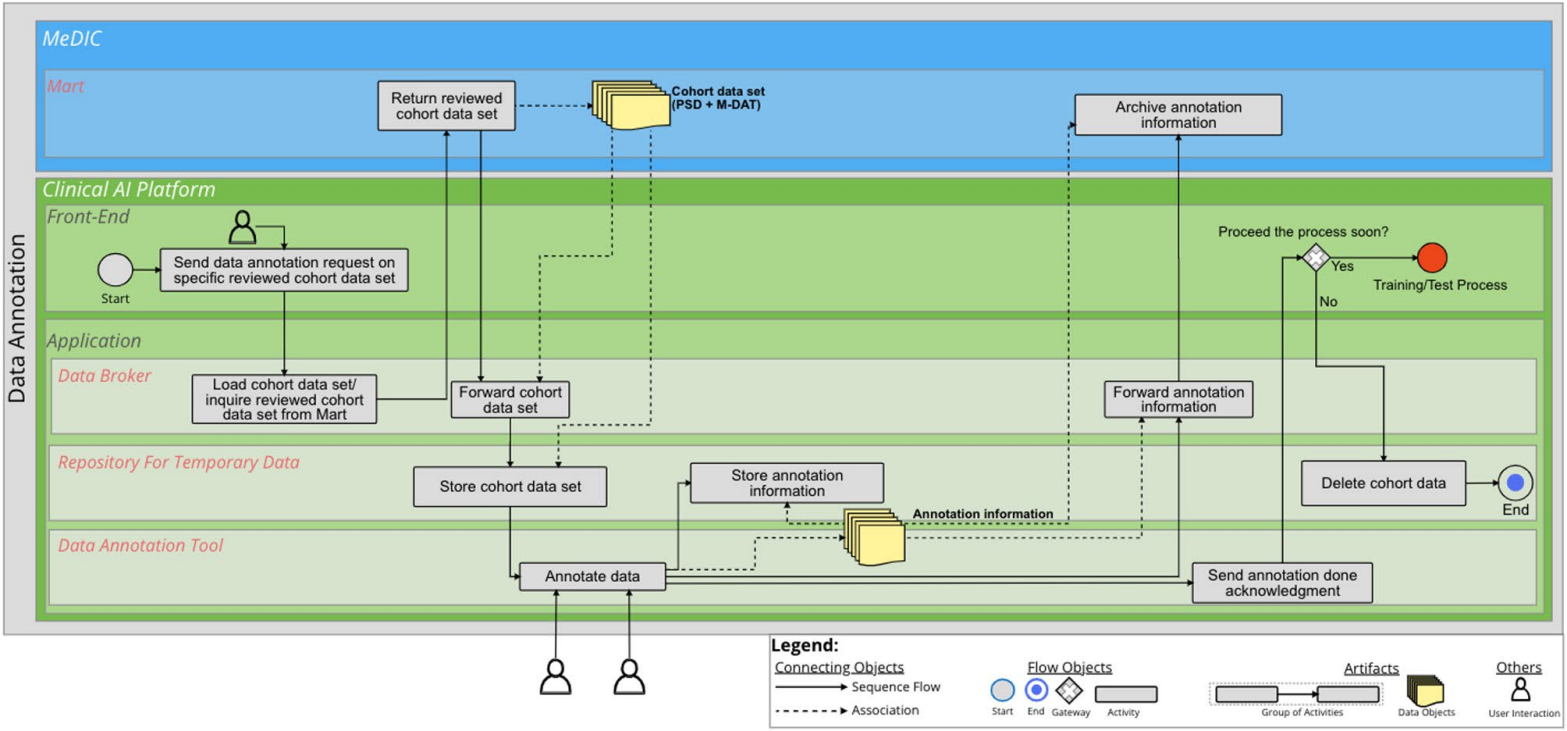
BPMN diagram for Data Annotation. Two IT system landscapes are illustrated here as sub-pools: the MeDIC and the Clinical AI Platform. For the MeDIC, the lane for the *Mart* and the lanes for the *Front-End* and *Application* of the Clinical AI Platform are shown here. The technical sub-components of *Data Broker*, *Data Annotation Tool* and the *Repository for Temporary Data* are relevant in the *Application* lane for this process

The second process describes the Data Annotation. The BPMN diagram for Data Annotation is illustrated in Fig. 2. An additional file shows the diagram in a higher resolution [see Additional file 2]’. We assume that the cohort data set is not available in the *Repository for Temporary Data*. The user sends a request from the *Front-End* for a specific, already reviewed and defined cohort data set to be annotated. The *Data Broker* forwards the request to the MeDIC *Mart*, which returns the pseudonymized medical data (PSN and MDAT). The retrieved cohort data are passed through the *Data Broker* to the *Repository for Temporary Data*. Once the cohort data are in the repository, they can be annotated by domain experts. When the annotation process is complete, the annotation information for the specific cohort data set is passed to MeDIC via the *Data Broker* and archived in the *Mart*. Once it is confirmed that the annotation process is completed, a decision is made by the User in the *Front-End* as to whether the cohort data can be further used in a timely manner or whether it can be deleted from the *Repository for Temporary Data*. If the process is to be continued in the near future, the next process would be On-Site Training and Testing. In such a case, the cohort data would remain in the *Repository for Temporary Data*.

### On-site Training and Testing

**Fig. 3:**
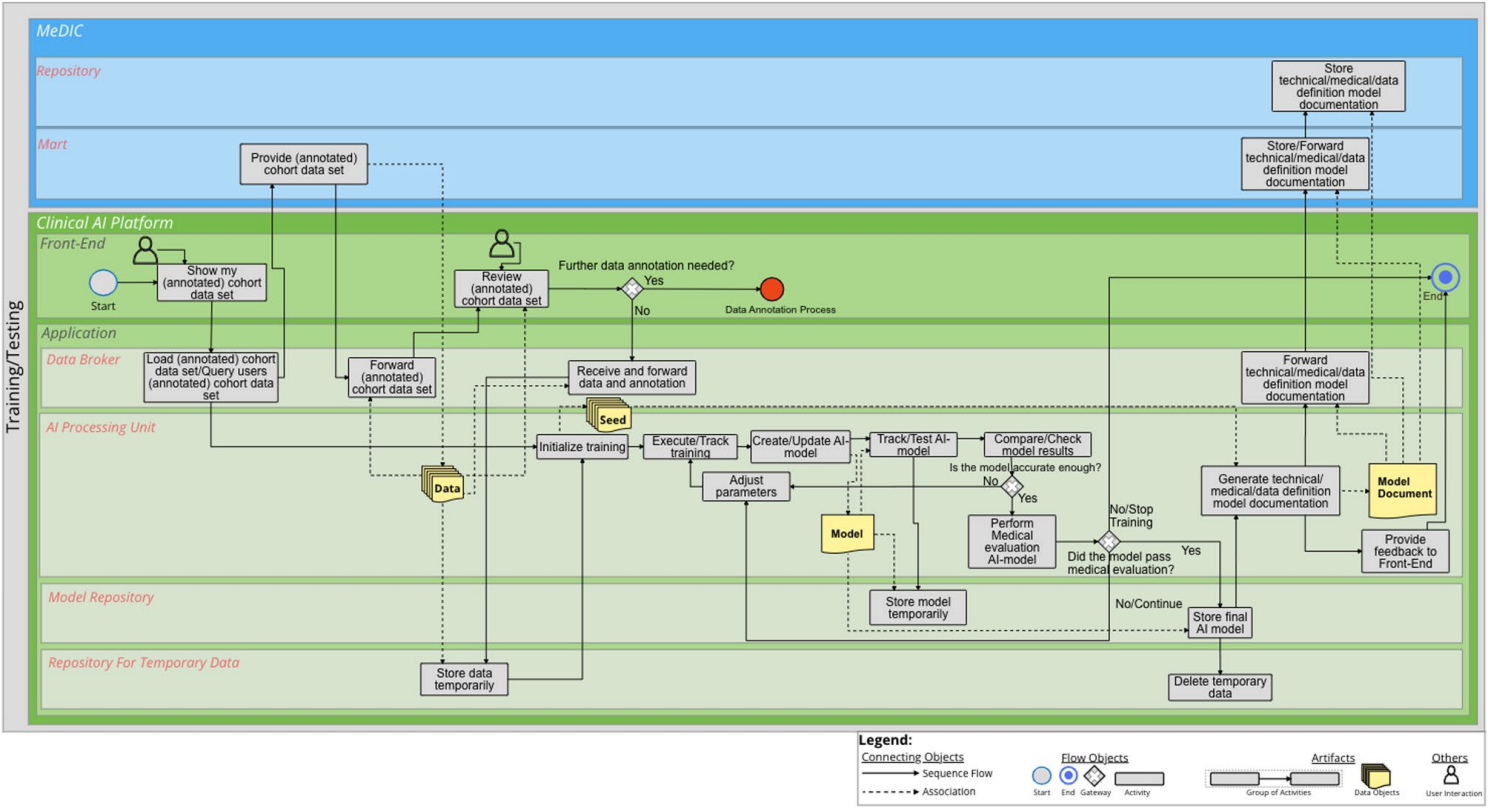
BPMN diagram for On-site Training and Testing. Two IT system landscapes are illustrated here as sub-pools: the MeDIC and the Clinical AI Platform. For the MeDIC, the lane for the *Mart* and *Repository* and the lanes for the *Front-End* and *Application* of the Clinical AI Platform are shown here. The technical sub-components of *Data Broker*, *AI Processing Unit*, *Model Repository* and the *Repository for Temporary Data* are relevant in the *Application* lane for this process

The third process is On-site Training and Testing. Figure 3 illustrates the BPMN diagram for On-site Training and Testing. An additional file shows the diagram in a higher resolution [see Additional file 3]’. We assume, that the cohort data set is not available in the *Repository for Temporary Data*. The user of the *Front-End* starts the process by retrieving the (annotated) cohort data set from the *Mart* via the *Data Broker*. The cohort data set is then reviewed by the user whether any further annotation is required. If further annotation is required, the annotation process is run again. If no further annotation is required, the *Data Broker* transfers the (annotated) cohort data set to the *Repository for Temporary Data*. Then the training of the AI model is initialized and performed in the *AI Processing Unit*. Note that a seed must be initialized for traceability of the training process. After each training session, the AI model is created or updated. The AI model is then tested and tracked. Once the AI model has been tested, it is checked to see whether the results obtained are satisfactory from a technical point of view. If this is not the case, the hyperparameters are adjusted and the training loop is run again. In the course of training, the AI model is temporarily stored in the *Model Repository*. After completion of the technical evaluation, a medical evaluation is performed. This medical evaluation includes, firstly, the clinician’s/user’s view of medical usability and relevance, and secondly, the regulatory evaluation, such as the Medical Device Regulation (MDR) [37]. If the result of the medical evaluation is negative, the training can be continued with adjusted parameters or the training can be stopped. If the medical evaluation is positive, the final AI model is either stored permanently in the *Model Repository* or, if other storage options exist (e.g. in the MeDIC), the AI model is stored elsewhere. This depends on the individual IT system design. Once the storage process of the AI model is completed, the data are deleted from the *Repository for Temporary Data*. Then a technical, medical and data definition documentation is created and stored in the *Mart* and *Repository* of the MeDIC.

### Inference

The fourth process is Inference. The Inference process is responsible for assessments based on incoming data. In this context, we distinguish between (Semi-Automated) Batch Inference and Real-Time Inference.

### (Semi-Automated) Batch Inference

**Fig. 4:**
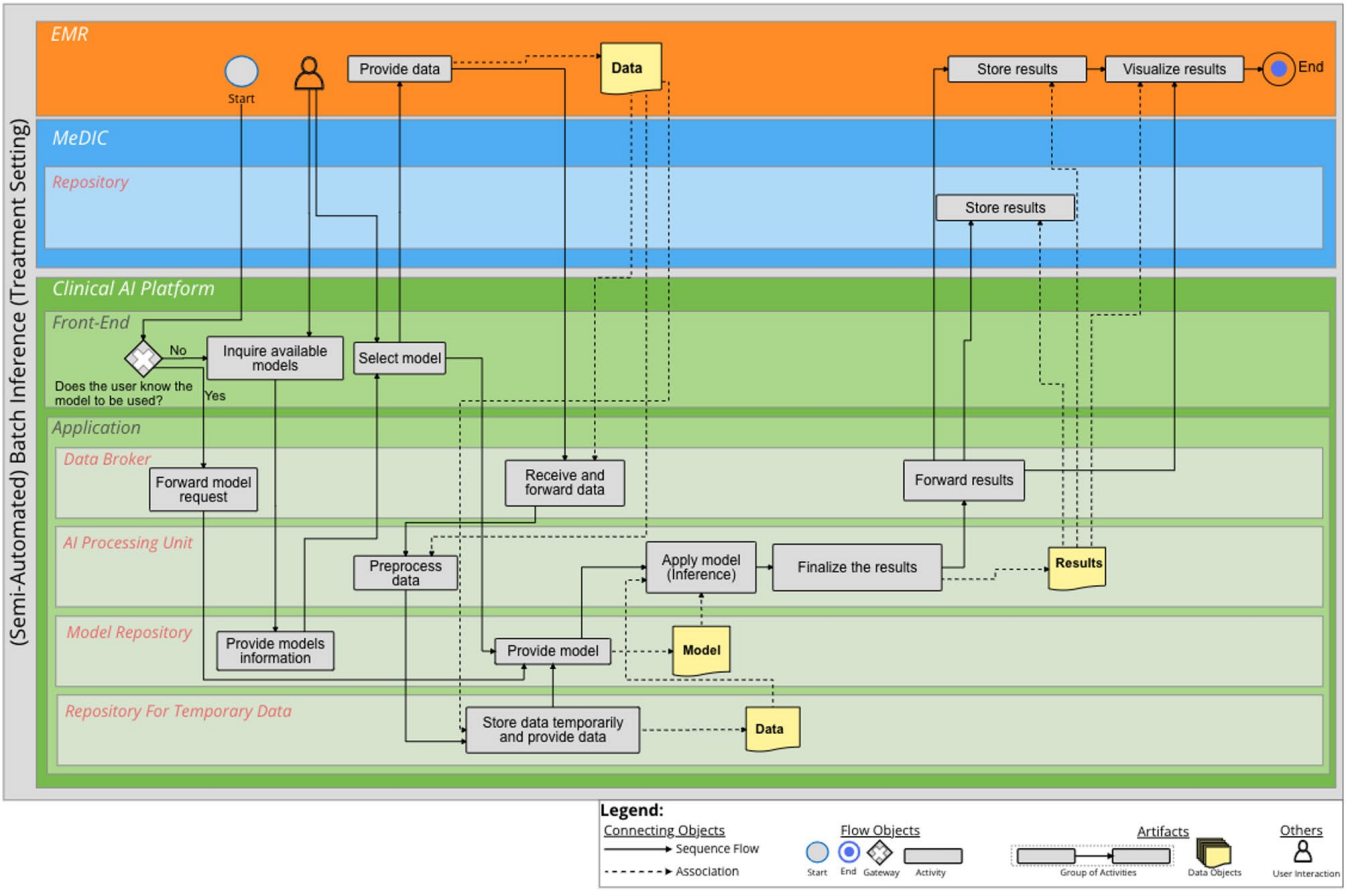
BPMN diagram for (Semi-Automated) Batch Inference (treatment setting). Three IT system landscapes are illustrated here as sub-pools: the EMR, the MeDIC and the Clinical AI Platform. For the MeDIC, the lane for the *Repository* and the lanes for the *Front-End* and *Application* of the Clinical AI Platform are shown here. The technical sub-components of *Data Broker*, *AI Processing Unit*, *Model Repository* and the *Repository for Temporary Data* are relevant in the *Application* lane for this process

The BPMN diagram for (Semi-Automated) Batch Inference is illustrated in Fig. 4. An additional file shows the diagram in a higher resolution [see Additional file 4]’. In this case, we are dealing with the treatment setting, which means that clinicians want to calculate a prediction for a batch. If the AI model to be used is already known, this process can be referred to as Semi Automated Batch Inference. The purpose of (Semi-Automated) Batch Inference is that data can be processed individually per data subset, i.e. patient. For this purpose, the user accesses the *Front-End* via a connected EMR system. After the available models are queried from the *Model Repository*, the user can select a suitable AI model if the AI model to be used is not yet known. The corresponding batch is then transferred from the EMR system to the *Repository for Temporary Data* of the Clinical AI Platform via the *Data Broker*, once preprocessing and a check of the data were successful. The selected model is loaded from the *Model Repository* into the *AI Processing Unit*. After the inference is performed successfully, the results are finalized for storage and visualization. The *Data Broker* transfers the results to the MeDIC *Mart*. Additionally, the results are transferred to the EMR. Finally, the results of the prediction are visualized in the EMR system.

**Fig. 5:**
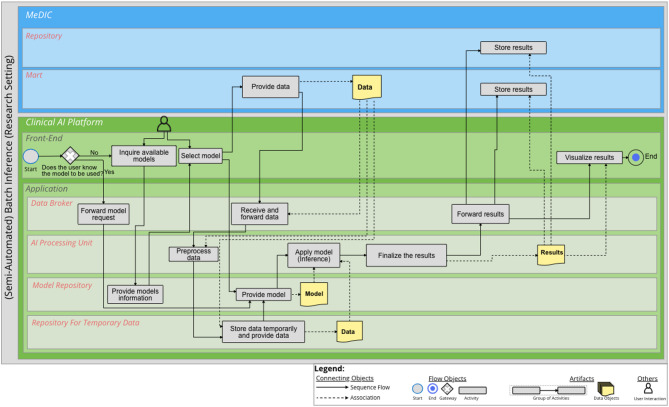
BPMN diagram for (Semi-Automated) Batch Inference (research setting). Two IT system landscapes are illustrated here as sub-pools: the MeDIC and the Clinical AI Platform. For the MeDIC, the lane for the *Mart* and *Repository* and the lanes for the *Front-End* and *Application* of the Clinical AI Platform are shown here. The technical sub-components of *Data Broker*, *AI Processing Unit*, *Model Repository* and the *Repository for Temporary Data* are relevant in the *Application* lane for this process

However, it is important to consider that the (Semi-Automated) Batch Inference can also be performed in a research setting. This scenario is shown in Fig. 5. An additional file shows the diagram in a higher resolution [see Additional file 5]’. In this case, researchers want to calculate a prediction for a batch. The difference from the treatment setting is that the researcher starts the inference within the Clinical AI Platform. In addition, the data are provided from the MeDIC *Mart* and not from the EMR. In this case, the results are stored in the *Mart* and depending on whether permanent storage is intended, also in the *Repository*. The visualization takes place in the Clinical AI Platform’s *Front-End*.

## Real-Time Inference

**Fig. 6:**
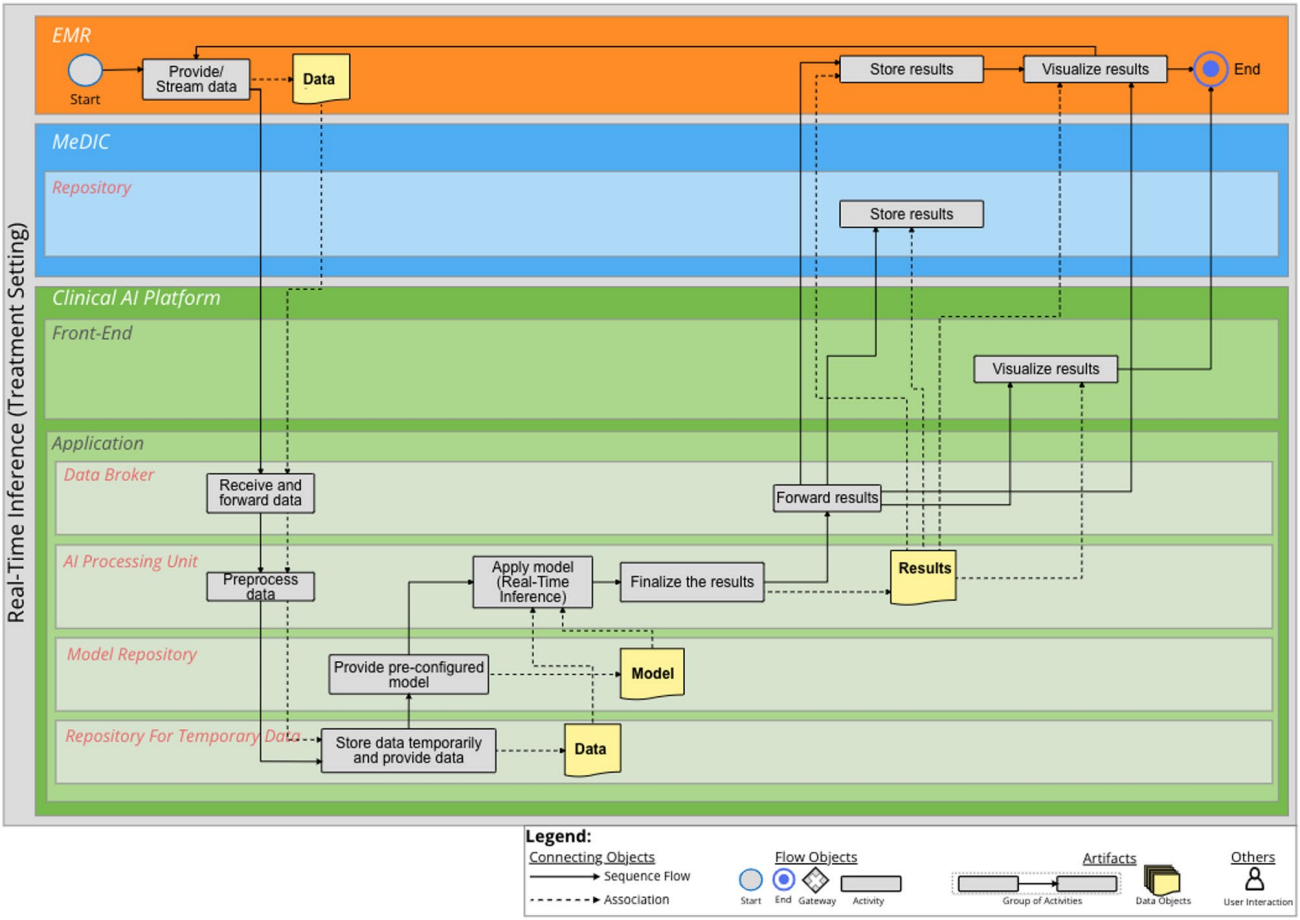
BPMN diagram for Real-Time Inference. Three IT system landscapes are illustrated here as sub-pools: the EMR, the MeDIC and the Clinical AI Platform. For the MeDIC, the lane for the *Repository* and the lanes for the *Front-End* and *Application* of the Clinical AI Platform are shown here. The technical sub-components of *Data Broker*, *AI Processing Unit*, *Model Repository* and the *Repository for Temporary Data* are relevant in the *Application* lane for this process

For the Real-Time Inference, it is also clear from the setting which model will be used. In this case, we assumed that only the treatment setting is relevant for the Real-Time Inference. A practical example is streaming data from the monitoring in the intensive care unit. Here, it is necessary to make real-time predictions from incoming streaming data. Apart from this, the process of providing the data and storing the results is similar to the inferences in the Figs. 4 and 5. The BPMN diagram for Real-Time Inference is illustrated in Fig. 6. An additional file shows the diagram in a higher resolution [see Additional file 6]’.

### Evaluation

The first use case for evaluation is the “digital x-ray assistant” from the KI-SIGS project. Initially, we start with the Data Selection process. We assume that the radiographs are provided via a MeDIC *Mart*. Accordingly, the cohort can be accessed by an authorized clinician via the *Front-End* of the Clinical AI Platform. The cohort is stored in the *Repository for Temporary Data*. The clinician can then evaluate, review and subsequently define and configure the cohort in the *Front-End* of the Clinical AI Platform. This includes checking that the existing images are usable. The configured cohort is forwarded to the *Repository for Temporary Data* via the *Data Broker* and stored there, while the initial cohort is deleted. The information about the configured cohort is also stored in the MeDIC *Mart*. We assume that the process will not be continued in a timely manner in this case, so that the cohort with the images will be deleted from the *Repository for Temporary Data*. Subsequently, the Data Selection process ends.

The next step is the Data Annotation process. Here, the user can query the already configured cohort via the *Front-End* of the Clinical AI Platform. The cohort is then loaded from the MeDIC *Mart* into the *Repository for Temporary Data* via the *Data Broker*. The cohort can afterwards be annotated using a linked data annotation tool. After the annotation is completed, the annotation information will be stored in the *Repository for Temporary Data* and also in the MeDIC *Mart* via the *Data Broker*. Since we assume in this case that there is a longer time delay after the annotation process, the annotation information is deleted from the *Repository for Temporary Data*.

Then the On-Site Training and Testing process starts. Once again, the user can retrieve the cohort, including the annotation from the MeDIC *Mart* in the *Front-End* of the Clinical AI Platform via the *Data Broker*. The user can check whether the cohort was already completely annotated or whether further annotation is necessary. Assuming that the annotation was already completed, the cohort is stored in the *Repository for Temporary Data*. Afterwards, the training can be initialized in the *AI Processing Unit*. A seed is defined, which is necessary for reproducibility. After the training is complete, an AI model is created, tested and then stored in the *Model Repository*. The AI model can then be checked with the help of various metrics. If, from a technical point of view, the results are not yet satisfactory or if further configurations to the CNN need to be evaluated, the training is started again. After the training is completed, the AI models can be compared to each other. If the technical evaluation of the AI model is successful, a medical evaluation can be performed to check the medical plausibility of the AI model. If this is successfully completed, the final AI model can be stored in the *Model Repository*. The temporarily stored data of the cohort in the *Repository for Temporary Data* are then deleted. Furthermore, the *AI Processing Unit* produces the technical, medical and data definition documentation. This documentation is stored in the MeDIC *Mart* and, if required, also in the MeDIC *Repository* for permanent storage. The On-site Training and Testing process is then completed.

In the following, the inference takes place in a treatment setting. Batch inference is used here, i.e. the clinician has a batch of radiographs on which a quality assessment of the upper ankle joint is to be performed. For this, the clinician can make a request for the available AI models via the *Front-End* of the Clinical AI Platform. The matching AI models are then forwarded from the *Model Repository* to the *Front-End* of the Clinical AI Platform. The clinician can select the appropriate model and simultaneously transfer his batch from the EMR to the Clinical AI Platform. The *AI Processing Unit* then performs the inference. The process is recorded and the results are stored and displayed in the EMR. For permanent storage, results can also be stored in the MeDIC *Repository*.

The second use case for the evaluation is the differentiation of benign from malignant tumors in breast cancer. We evaluate whether our BPMN diagrams are effective for Data Selection, On-Site Training and Testing and Semi-Automated Batch Inference within the research setting. In this case, we assume once again that the user is authorized by the responsible Use & Access Committee to receive the cohort data set with the Wisconsin Breast Cancer data set. Accordingly, the user sends a request from the *Front-End* through the *Data Broker* to retrieve the pseudonymized cohort data set, which is already made available via a MeDIC *Mart*. Subsequently, the cohort data set is stored in the *Repository for Temporary Data*. Once the cohort data set is available in the Clinical AI Platform, the user can evaluate, review and define the cohort in the *Front-End*. Afterwards, the information about the reviewed cohort data set is stored both in the *Repository for Temporary Data* and in the MeDIC *Mart*. In this case, the process is continued right away, so the data cohort can remain in the *Repository for Temporary Data* for now.

As the data are already annotated, we continue with the On-Site Training and Testing process. We move to the initialization of training, as the cohort data set already exists in the *Repository for Temporary Data*. A seed is defined, which is necessary for reproducibility. Then the training is performed and tracked. The process of training and testing is performed similarly to the process of the first use case. Once the AI model has passed medical evaluation, the final AI model is stored in the Model Repository. Subsequently, the cohort data of the Wisconsin Breast Cancer data set can be deleted from the *Repository for Temporary Data*. At the same time, technical and medical documentation are generated and then stored in the MeDIC *Mart* and/or the MeDIC *Repository* via the *Data Broker*.

In the following, the researchers want to apply inference based on a new cohort that contains parameters describing the cell nucleus from images. In this case, the Semi-Automated Batch Inference in the research setting is applied. The user activates the AI model for the given use case for the classification of benign and malignant breast tumors via the *Front-End*. The AI model is loaded into the *AI Processing Unit*. Simultaneously, the cohort data set for the inference will be retrieved from the MeDIC *Mart* and loaded to the *Repository for Temporary Data* via the *Data Broker*. After the decision tree model from the *Model Repository* and the new cohort from the MeDIC *Mart* are loaded into the *AI Processing Unit*, the inference is performed. The results are processed and stored in the MeDIC *Mart* and, if necessary, in the MeDIC *Repository*. The results are then visualized in the *Front-End* of the AI platform.

The third use case is “Risk indicators for cardiopulmonary decompensation in intensive care units by monitoring vital signs”. Upon approval by the Use & Access Committee to use the cohort data set and its provision in the MeDIC *Mart*, the user can request the cohort data set in the *Front-End* via the *Data Broker* from the MeDIC *Mart*. The cohort is then stored in the *Repository for Temporary Data*. The user can evaluate, review and define the cohort in the *Front-End* once the cohort data set is available in the Clinical AI Platform. This involves evaluating whether the data are fit for purpose or they are flawed or incomplete, and thus unusable. Afterwards, the configured cohort is stored via the *Data Broker* in the *Repository for Temporary Data* and in the MeDIC *Mart*. Since the cohort data set requires annotation, we proceed to the annotation process. The user can retrieve the cohort from the *Repository for Temporary Data* in the *Front-End* and annotate the data. The annotation consists of calculating Decompensation Scores (DEC scores), which provide information on the timing of the relevant occurrences. A distinction is made between no decompensation, beginning-moderate decompensation and severe decompensation. These classes should be predicted up to 24 h before occurrence. As soon as the annotation is completed, the annotation information is stored in the *Repository for Temporary Data* and via *Data Broker* also in the MeDIC *Mart*. We assume that there is a longer time delay between the data annotation process and the On-site Training and Testing process. Therefore, the cohort is deleted from the *Repository for Temporary Data*.

As soon as the On-site Training and Testing process is started, the user can retrieve the cohort in the *Front-End* from the MeDIC *Mart* via *Data Broker*. The process of training and testing is performed similarly to the process of the other two use cases. The On-site Training and Testing process ends with feedback to the Front-End, if the process has been executed successfully.

In this use case, the continuous processing of vital signs is crucial for the early detection of cardiopulmonary decompensation. For this purpose, the EMR system continuously delivers the vital signs to the TKIP via the *Data Broker*. To ensure correct processing, the incoming data are preprocessed in the *AI Processing Unit*. This includes the selection of the required parameters with the correct unit. The data are temporarily stored in the *Repository for Temporary Data* and then the trained AI model is loaded from the *Model Repository* to the *AI Processing Unit*. There, the data are loaded from the *Repository for Temporary Data* and then *Real-Time Inference* is performed. The results are then processed for presentation, which includes the classification of the DEC score. The *Data Broker* forwards the results to the EMR and the MeDIC *Repository*, enabling the results to be displayed in the EMR. Optionally, the results can also be displayed in the *Front-End* of the Clinical AI Platform. The next data set from the EMR system is then prepared for Real-Time Inference, resulting in the recurrence of the procedure.

## Discussion

### Principal results

The aim of this work was to demonstrate the integration of a clinical AI platform into clinical IT, including the entire development cycle for an AI model using BPMN process diagrams. For this purpose, the requirements determined in the sub-project “Technical AI Platform” of KI-SIGS were used as a basis to define the boundary conditions for the development of the BPMN diagrams. The processes of the BPMN diagrams were determined on the basis of literature. The presented BPMN diagrams cover the entire development cycle of an AI model with the differentiated processes of Data Selection, Data Annotation, On-site Training and Testing, Batch Inference, Semi-Automated Batch Inference and Real-Time Inference. The three IT system landscapes at the UKSH are of particular importance for this purpose: EMR, MeDIC and the Clinical AI Platform. We identified two components of MeDIC that are relevant in our development context: *Mart* and *Repository*. The Clinical AI Platform consists of a *Front-End* and an *Application* part. The *Application* consists of a *Data Broker*, which is necessary for communication between the IT system landscapes, an *AI Processing Unit*, which processes all activities for which dedicated computing capacity is required. The application also includes a *Model Repository* for the AI models and a *Repository for Temporary Data* for the temporary storage of cohorts. Other tools can also be integrated into the Clinical AI Platform for data annotation. The BPMN diagram for Data Selection shows how the cohorts provided in the MeDIC *Mart* can be retrieved and defined by the user in the Clinical AI Platform. The BPMN diagram of Data Annotation demonstrates the process of how the cohort data set can be annotated in the Clinical AI Platform and can then be stored with the cohort itself. The BPMN diagram of On-site Training and Testing displays how an already defined and annotated cohort data set can be loaded and an already defined AI algorithm can be trained with this cohort. We distinguish between three different cases for inference to cover the spectrum of possible clinical use cases: Batch Inference, Semi-Automated Batch Inference and Real-Time Inference. The Batch Inference describes the case of Inference for single or multiple cohort data instances with manual AI model selection. Semi-Automated Batch Inference also describes inference with single or multiple cohort data instances, but with a pre-selected AI model that can automatically be retrieved. Real-Time Inference describes the inference of streaming data that can, e.g., be observed in ICU use cases for monitoring patients’ vital parameters. We evaluated these processes with three clinical use cases that verify the completeness of the BPMN diagrams. We selected the FEDS framework for evaluating the BPMN diagrams to ensure a systematic analysis and assessment of our results. The FEDS framework also ensures a consistent and comparable evaluation [31]. With the aid of three independent clinical use cases, we were able to demonstrate for each individual process, as well as for the overall evaluation objective, that the AI development cycle can be successfully represented with the developed processes, thus minimizing technical uncertainty. Therefore, it can be determined that the processes represent the complete and comprehensive development cycle of an AI model within clinical IT, according to Lu et al. [28].

### Limitations

Although our BPMN diagrams map a broad spectrum of AI development in a clinical context, there may be use cases that require a more specific process mapping. There may be individual use cases that require more human interaction, thus leading to multiple iterations. Accordingly, the processes and the corresponding BPMN diagrams can be considered as blueprints that are suitable for most clinical use cases. Furthermore, it is important to consider the depth of the process mapping. Many activities within the processes can certainly be displayed in more detail. Accordingly, we have chosen the trade-off in favor of generalizability rather than depth of detail in order to map the range of clinical use cases as accurately as possible. Another aspect is the consideration of security and privacy factors. This would be a useful extension of the BPMN process diagrams. Non-functional aspects such as security or privacy factors were implicitly considered in our elaborations because the priority was to consider and implement the functional aspects to demonstrate the functionalities of the Clinical AI Platform. The privacy by design principles, according to Cavoukian et al. [38] were recognized in general. However, the explicit consideration of privacy and security factors requires a separate and more detailed assessment. In general, privacy by design should be ensured for the clinical AI platform. In addition, the prevailing data protection regulations should always be taken into account. Appropriate authentication and authorization mechanisms for different user groups should also be implemented to ensure an adequate level of data security. It is also important to ensure that the costs of implementing, integrating and operating a platform are in proportion to the benefits of the platform. Further development of the BPMN diagrams could also include consideration of paradigms such as federated learning, which includes the aggregation of cohort data sets from multiple sites. This would ensure greater data security and privacy if data does not have to be exchanged for training. It would also prevent huge amounts of data from being transferred and thus a redundancy of data. In our BPMN diagrams, the exchange of submodels and the aggregation of those would have to be mapped. In our paper, the training/testing scenario only referred to On-site Training and Testing.

### Comparison with prior work

Previous publications have shown which processes exist within the development cycle of an AI model [27, 28]. While Amershi et al. [27] show the development cycle of an AI model in nine small steps, Lu et al. [28] present a more summarized development cycle in six steps. In our approach, we have summarized the development cycle of Lu et al. [28] even further and presented the entire development cycle of an AI model in only four overarching steps. Our approach focuses on the technical development cycle and excludes preparatory processes that are usually not specific to the development of AI models. The main advantage of our approach is the comprehensibility of the technical development process while covering generic clinical AI use cases. Amershi et al. focus on AI model development without specific emphasis on any particular domain, while Lu et al. describe the processes they have identified in the clinical domain but rather generally. In contrast to this, we describe the processes that we have identified in detail with the help of BPMN diagrams, which is a novel approach in the field of clinical AI model development. Previous work also shows possible integrations of an AI platform with clinical IT [14–16]. However, they are either limited to certain data types or to medical domains. While Gruendner et al. [14] only present the integration of data with FHIR, Scherer et al. [15] and Leiner et al. [16] only focus on imaging data. In our approach, there is no limitation in terms of data types. Our approach addresses the entirety of clinical data types. Besides, Gruendner et al., Scherer et al. and Leiner et al. [14–16] provide no description of the development of relevant AI processes in the hospital, which include the medical data integration centers and the primary systems. In this study, we demonstrate how our main functional requirements can be leveraged as boundary conditions to describe the processes for development and deployment of clinical AI models with BPMN diagrams. This provides a foundation for the future development of a clinical AI platform architecture.

## Conclusions

With the BPMN diagrams presented in this paper, we laid the foundation to design the architecture of a clinical AI platform. The architecture and implementation of the AI platform in KI-SIGS will be based on the BPMN diagrams. In addition, the processes and interaction between the three clinical IT system landscapes of the EMR, the MeDIC and the Clinical AI platform are shown for the first time. With the help of the use case agnostic processes presented, the entire workflow for creating and applying a clinical AI model can be illustrated. Our BPMN diagrams also provide a basis for similar projects to develop an AI platform. In many cases, clinical AI platforms can be realized that are based on an abstracted version of our BPMN diagrams.

## Electronic supplementary material

Below is the link to the electronic supplementary material.

**Supplementary Material 1:** Additional file 1 (PDF) - BPMN diagram for Data Selection. Two IT system landscapes are illustrated here as sub-pools: the MeDIC and the Clinical AI Platform. For the MeDIC, the lane for the *Mart* and the lanes for the *Front-End* and *Application* of the Clinical AI Platform are shown here. The technical sub-components of *Data Broker* and the *Repository for Temporary Data* are relevant in the *Application* lane for this process

**Supplementary Material 2:** Additional file 2 (PDF) - BPMN diagram for Data Annotation. Two IT system landscapes are illustrated here as sub-pools: the MeDIC and the Clinical AI Platform. For the MeDIC, the lane for the *Mart* and the lanes for the *Front-End* and *Application* of the Clinical AI Platform are shown here. The technical sub-components of *Data Broker*, *Data Annotation Tool* and the *Repository for Temporary Data* are relevant in the *Application* lane for this process

**Supplementary Material 3:** Additional file 3 (PDF) - BPMN diagram for On-site Training and Testing. Two IT system landscapes are illustrated here as sub-pools: the MeDIC and the Clinical AI Platform. For the MeDIC, the lane for the *Mart* and *Repository* and the lanes for the *Front-End* and *Application* of the Clinical AI Platform are shown here. The technical sub-components of *Data Broker*, *AI Processing Unit*, *Model Repository* and the *Repository for Temporary Data* are relevant in the *Application* lane for this process

**Supplementary Material 4:** Additional file 4 (PDF) - BPMN diagram for (Semi-Automated) Batch Inference (treatment setting). Three IT system landscapes are illustrated here as sub-pools: the EMR, the MeDIC and the Clinical AI Platform. For the MeDIC, the lane for the *Repository* and the lanes for the *Front-End* and *Application* of the Clinical AI Platform are shown here. The technical sub-components of *Data Broker*, *AI Processing Unit*, *Model Repository* and the *Repository for Temporary Data* are relevant in the *Application* lane for this process

**Supplementary Material 5:** Additional file 5 (PDF) - BPMN diagram for (Semi-Automated) Batch Inference (research setting). Two IT system landscapes are illustrated here as sub-pools: the MeDIC and the Clinical AI Platform. For the MeDIC, the lane for the *Mart* and *Repository* and the lanes for the *Front-End* and *Application* of the Clinical AI Platform are shown here. The technical sub-components of *Data Broker*, *AI Processing Unit*, *Model Repository* and the *Repository for Temporary Data* are relevant in the *Application* lane for this process

**Supplementary Material 6:** Additional file 6 (PDF) - BPMN diagram for Real-Time Inference. Three IT system landscapes are illustrated here as sub-pools: the EMR, the MeDIC and the Clinical AI Platform. For the MeDIC, the lane for the *Repository* and the lanes for the *Front-End* and *Application* of the Clinical AI Platform are shown here. The technical sub-components of *Data Broker*, *AI Processing Unit*, *Model Repository* and the *Repository for Temporary Data* are relevant in the *Application* lane for this process

## Data Availability

The data used in the current study are not publicly available but are available from the corresponding author upon reasonable request.

## Footnotes

1 Funded by the German Federal Ministry of Education and Research.

## Abbreviations

A&I: Application & Innovation
AI: Artificial Intelligence
API: Application Programming Interface
BPMN: Business Process Model and Notation
CNN: Convolutional Neural Network
DEC scores: Decompensation scores
DIZ: Data Integration Center
DSR: Design Science Research
EDC: Electronic Data Capture
EMR: Electronic Medical Record
FEDS: Framework for Evaluation in Design Science
FHIR: Fast Healthcare Interoperability Resource
GRU: Gated Recurrent Unit
ICU: Intensive Care Unit
IT: Information Technology
MDAT: Medical Data
MDR: Medical Device Regulation
MeDIC: Medical Data Integration Center
ML: Machine Learning
PACS: Picture Archiving and Communication System
PSN: Pseudonym
UKSH: University Hospital Schleswig-Holstein
