# Supplementary figures and images for: Integrating an AI platform into clinical IT: BPMN processes for clinical AI model development
Source: BMC Med Inform Decis Mak. 2025 Jul 2;25:243. doi: 10.1186/s12911-025-03087-4 (PMC12218938; doi:10.1186/s12911-025-03087-4)

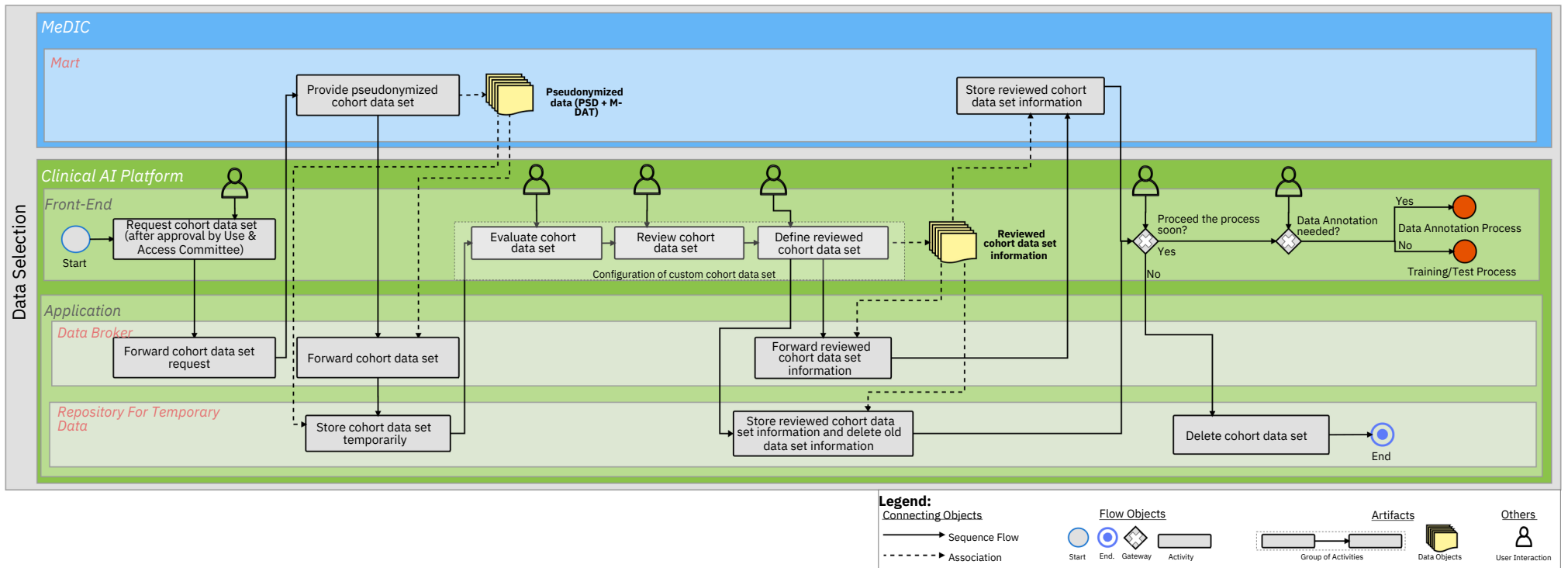

Supplement: Supplementary file 1 — Supplementary Material 1: Additional file 1 (PDF) - BPMN diagram for Data Selection. Two IT system landscapes are illustrated here as sub-pools: the MeDIC and the Clinical AI Platform. For the MeDIC, the lane for the Mart and the lanes for the Front-End and Application of the Clinical AI Platform are shown here. The technical sub-components of Data Broker and the Repository for Temporary Data are relevant in the Application lane for this process [file 12911_2025_3087_MOESM1_ESM.pdf]

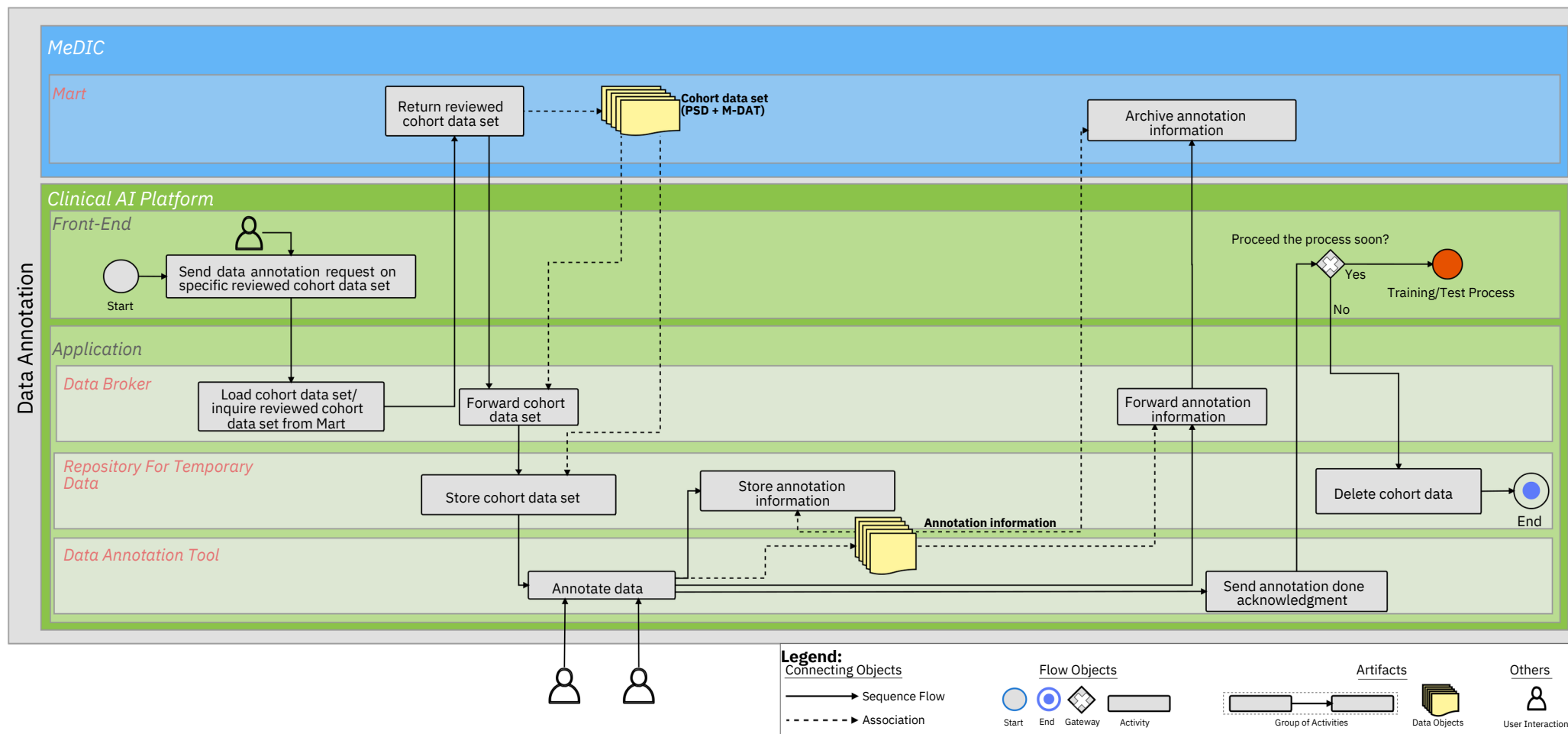

Supplement: Supplementary file 2 — Supplementary Material 2: Additional file 2 (PDF) - BPMN diagram for Data Annotation. Two IT system landscapes are illustrated here as sub-pools: the MeDIC and the Clinical AI Platform. For the MeDIC, the lane for the Mart and the lanes for the Front-End and Application of the Clinical AI Platform are shown here. The technical sub-components of Data Broker, Data Annotation Tool and the Repository for Temporary Data are relevant in the Application lane for this process [file 12911_2025_3087_MOESM2_ESM.pdf]

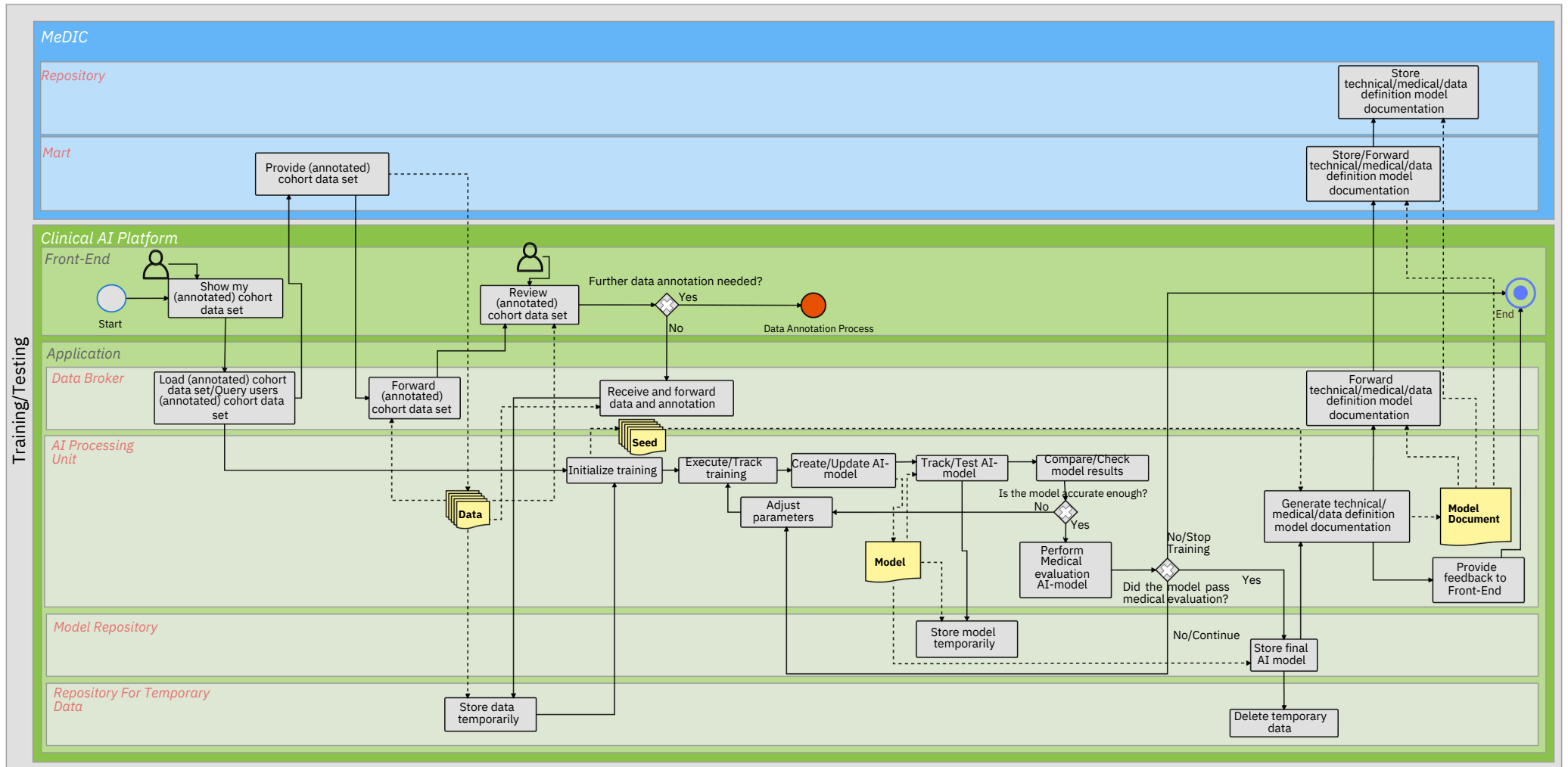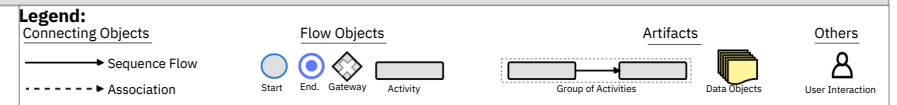

Supplement: Supplementary file 3 — Supplementary Material 3: Additional file 3 (PDF) - BPMN diagram for On-site Training and Testing. Two IT system landscapes are illustrated here as sub-pools: the MeDIC and the Clinical AI Platform. For the MeDIC, the lane for the Mart and Repository and the lanes for the Front-End and Application of the Clinical AI Platform are shown here. The technical sub-components of Data Broker, AI Processing Unit, Model Repository and the Repository for Temporary Data are relevant in the Application lane for this process [file 12911_2025_3087_MOESM3_ESM.pdf]

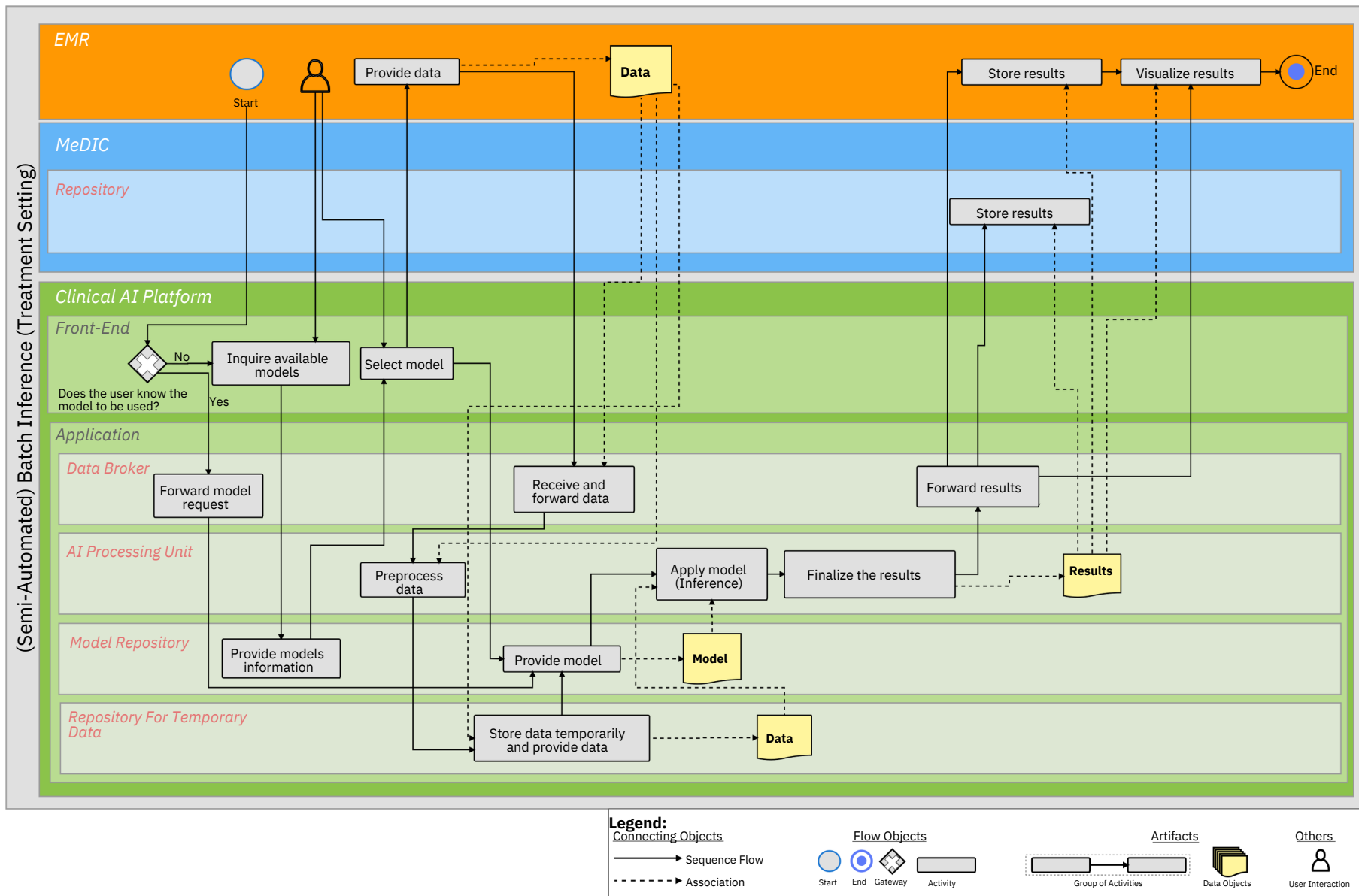

Supplement: Supplementary file 4 — Supplementary Material 4: Additional file 4 (PDF) - BPMN diagram for (Semi-Automated) Batch Inference (treatment setting). Three IT system landscapes are illustrated here as sub-pools: the EMR, the MeDIC and the Clinical AI Platform. For the MeDIC, the lane for the Repository and the lanes for the Front-End and Application of the Clinical AI Platform are shown here. The technical sub-components of Data Broker, AI Processing Unit, Model Repository and the Repository for Temporary Data are relevant in the Application lane for this process [file 12911_2025_3087_MOESM4_ESM.pdf]

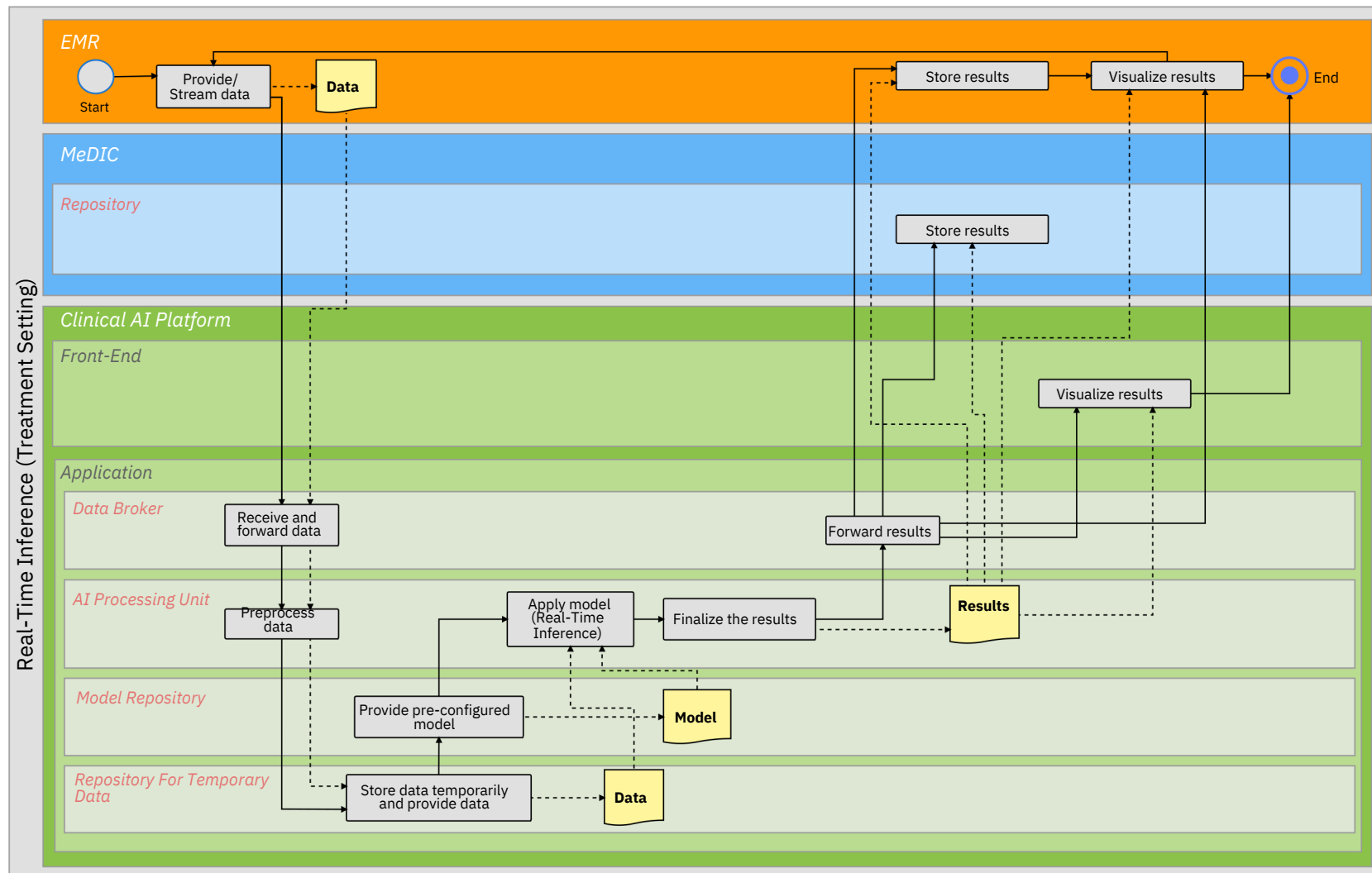

#### Legend:

##### Connecting Objects

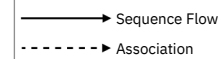

##### Flow Objects

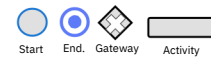

##### Artifacts

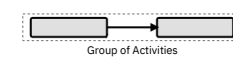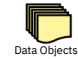

##### Others

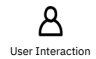

Supplement: Supplementary file 6 — Supplementary Material 6: Additional file 6 (PDF) - BPMN diagram for Real-Time Inference. Three IT system landscapes are illustrated here as sub-pools: the EMR, the MeDIC and the Clinical AI Platform. For the MeDIC, the lane for the Repository and the lanes for the Front-End and Application of the Clinical AI Platform are shown here. The technical sub-components of Data Broker, AI Processing Unit, Model Repository and the Repository for Temporary Data are relevant in the Application lane for this process [file 12911_2025_3087_MOESM6_ESM.pdf]
