# Supplementary material for: Integrating an AI platform into clinical IT: BPMN processes for clinical AI model development
Source: BMC Med Inform Decis Mak. 2025 Jul 2;25:243. doi: 10.1186/s12911-025-03087-4 (PMC12218938; doi:10.1186/s12911-025-03087-4)
Supplement: Supplementary file 5 — Supplementary Material 5: Additional file 5 (PDF) - BPMN diagram for (Semi-Automated) Batch Inference (research setting). Two IT system landscapes are illustrated here as sub-pools: the MeDIC and the Clinical AI Platform. For the MeDIC, the lane for the Mart and Repository and the lanes for the Front-End and Application of the Clinical AI Platform are shown here. The technical sub-components of Data Broker, AI Processing Unit, Model Repository and the Repository for Temporary Data are relevant in the Application lane for this process [file 12911_2025_3087_MOESM5_ESM.pdf]

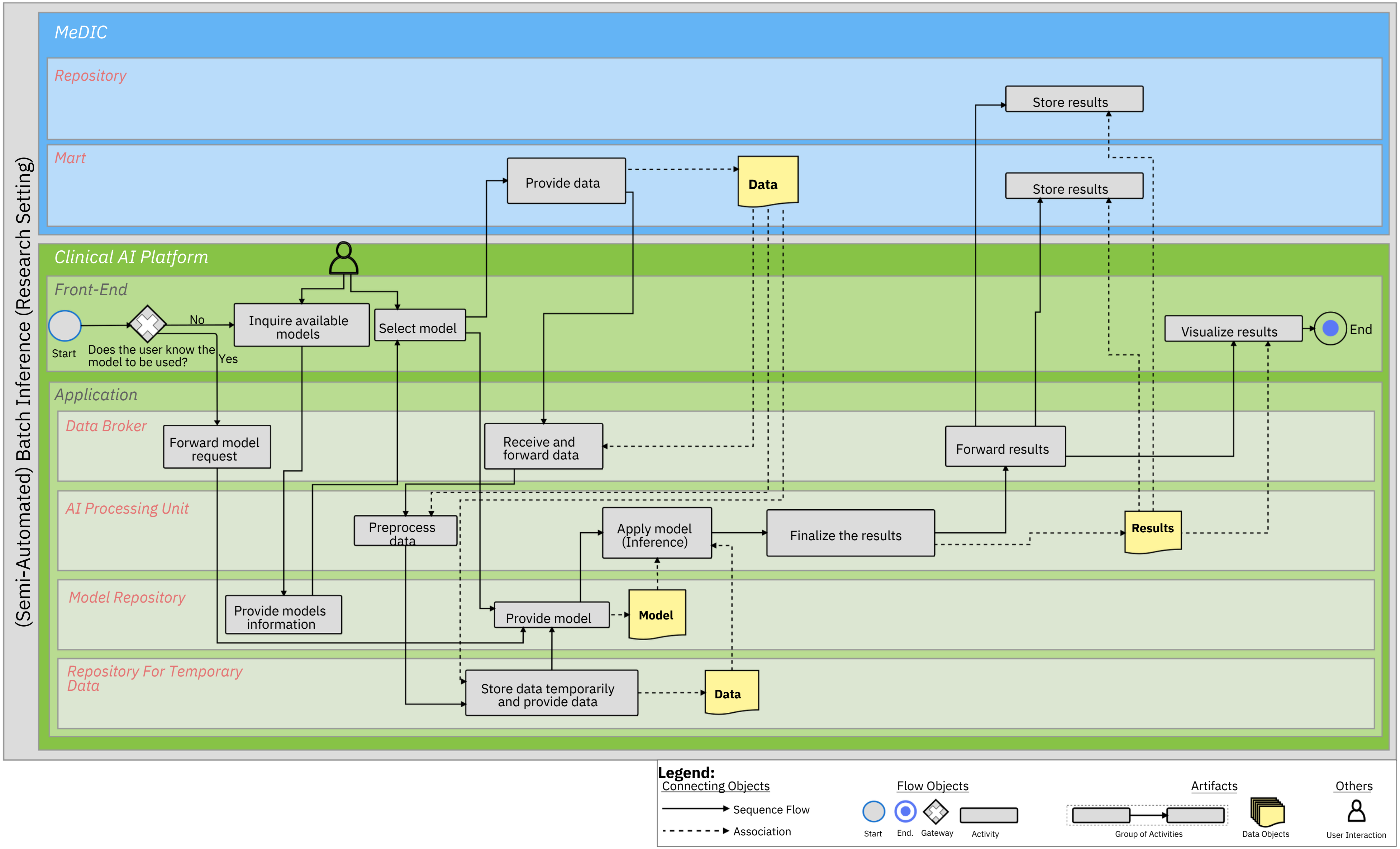
(Semi-Automated) Batch Inference (Research Setting)
MeDIC
Repository
Store results
Mart
Provide data
Data
Store results
Clinical AI Platform
Front-End
Start
Does the user know the model to be used?
No
Yes
Inquire available models
Select model
Visualize results
End
Application
Data Broker
Forward model request
Receive and forward data
Forward results
AI Processing Unit
Preprocess data
Apply model (Inference)
Finalize the results
Results
Model Repository
Provide models information
Provide model
Model
Repository For Temporary Data
Store data temporarily and provide data
Data
Legend:
Connecting Objects
Sequence Flow
Association
Flow Objects
Start
End.
Gateway
Activity
Artifacts
Group of Activities
Data Objects
Others
User Interaction
